# A polo-like kinase 1 inhibitor enhances erastin sensitivity in head and neck squamous cell carcinoma cells in vitro

**DOI:** 10.1007/s00280-024-04654-8

**Published:** 2024-03-27

**Authors:** Xiangping Wu, Jing Wu

**Affiliations:** Department of Otolaryngology-Head & Neck Surgery, The First Affiliated Hospital of Anhui Medical University, Hefei, 230022 Anhui China

**Keywords:** BI 2536, PLK1, Erastin, HNSCC

## Abstract

**Background:**

Polo-like kinase 1 (PLK1) is a critical therapeutic target in the treatment of head and neck squamous cell carcinoma (HNSCC). The objective of this study was to investigate the therapeutic effect of the combination of BI 2536, a PLK1 inhibitor, and erastin, a ferroptosis inducer, in HNSCC.

**Methods:**

The proliferation, invasion, and migration abilities of Tu177 and FaDu cells upon exposure to BI 2536 and erastin, used in combination or alone, were tested. Fe^2+^, glutathione (GSH), and malondialdehyde (MDA) detection kits were used to determine whether the addition of BI 2536 enhanced the accumulation of Fe^2+^ and MDA, along with the depletion of GSH. Quantitative real-time PCR, western blot analyses were performed to investigate whether BI 2536 further altered the mRNA and expression level of ferroptosis genes. Furthermore, si PLK1 was used to investigate whether targeting PLK1 gene promoted erastin-induced ferroptosis.

**Results:**

The combination of BI 2536 and erastin exerted a stronger cytotoxicity than treatment with a single agent. Compared with erastin treatment alone, the combination of BI 2536 and erastin lowered the ability of tumor cells to self-clone, invade, and migrate. BI 2536 enhanced the accumulation of Fe^2+^ and MDA, and the depletion of GSH. BI 2536 increased erastin-induced changes in ferroptosis-related gene mRNA and expression. Importantly, targeting PKL1 enhanced the anti-cancer effect of erastin.

**Conclusion:**

BI 2536 enhanced the sensitivity of HNSCC cells to erastin, which provides a new perspective for cancer treatment.

## Introduction

The global incidence rate and mortality of head and neck squamous cell carcinoma (HNSCC) is on the rise [1]. For patients with advanced tumors, chemotherapy is the first-line treatment, as recommended in the current clinical guidelines [2]. Among the chemotherapy treatment options, the therapeutic effect of combining agents is significantly higher than that of single-drug therapy [2, 3]. Various existing chemotherapy drugs mainly kill cancer cells by activating cell death pathways. Ferroptosis is a novel cell death pathway that has been widely studied and is considered a new direction for cancer treatment.

By definition, ferroptosis is a non-apoptotic form of death that relies on the accumulation of intracellular iron that increases the levels of toxic lipid peroxides, which are closely related to cellular metabolism [4]. Specifically, when intracellular and extracellular iron homeostasis pathways are disrupted, the accumulated Fe^2+^ in the cell triggers the Fenton reaction, causing an imbalance in the production and degradation of intracellular reactive oxygen species (ROS) [5]. Acyl-CoA synthetase long-chain family member 4 (ACSL4) is an essential enzyme that triggers an increase in lipid peroxidation by participating in the synthesis of easily oxidized membrane phospholipids [6, 7]. Another important feature of ferroptosis is a decrease in the activity of the glutathione (GSH) system’s core enzyme, glutathione peroxidase 4 (GPX4), which is directly related to a cellular redox imbalance [8]. Solute carrier family 7 member 11 (SLC7A11) is a component of the cystine/glutamate transporter system (Xc^−^) responsible for mediating the activity of cysteine/glutamic acid reverse transporters, thereby transporting the raw materials for synthesizing GSH [9, 10]. GPX4 uses GSH to reduce lipid hydroperoxides to lipid alcohols to defend against iron-mediated cell death. When the activity of system Xc^−^ is inhibited, GPX4 is inactivated due to a decrease in GSH synthesis, further disrupting the cellular antioxidant system [9].

In terms of cancer, ferroptosis directly kills tumor cells, overcomes both radiation and chemotherapy resistance, demonstrating enormous therapeutic potential in cancer research [11, 12]. However, the complex tumor microenvironment and unique pathological microenvironment of various tumor sites severely limit the therapeutic effect of drugs that elicit ferroptosis [13]; combination therapy may improve the therapeutic effect of such drugs.

Polo-like kinase 1 (PKL1) is a regulatory factor involved in the initiation, maintenance, and termination of cell mitosis, and is primarily responsible for promoting the G2/M phase transition and regulating DNA synthesis [14]. PKL1 is generally overexpressed in thyroid, laryngeal, breast, and colon cancers cancers [15–18], driving tumor metastasis, and drug resistance, and is a potentially important target in various cancer types [19]. In recent years, PLK1 inhibitors have been developed for single-drug or combination therapy. BI 2536 is a potent and selective PLK1 inhibitor. Its mechanism of reducing PLK1 activity is mainly by targeting the ATP binding domain [19, 20]. BI 2536 also exerts therapeutic effects on adrenocortical carcinoma by disrupting centrosome homeostasis and enhances the sensitivity of esophageal cancer to cisplatin by inducing apoptosis [21, 22]. The combination of BI 2536 and cisplatin shows high anti-tumor activity in gastric cancer [23]. Herein, we investigated the combined therapeutic effect of BI 2536 and the ferroptosis inducer, erastin, in HNSCC cells in vitro.

## Materials and methods

### Cell lines and reagents

Human laryngeal squamous cell carcinoma Tu177 cells, and human pharyngeal squamous cell carcinoma FaDu cells, were purchased from the Peking Union Cell Bank (Beijing, China). The culture medium was Dulbecco’s modified Eagle’s medium (Hyclone, Logan, UT, USA). Incubator conditions were 37 ℃, 95% humidity, and 5% CO_2_. BI 2536 (#HY-50698), erastin (#HY-15763) and Ferrostatin-1 (#HY-100579) were purchased from MedChemExpress (Monmouth Junction, NJ, USA).

### Cell viability assay

Briefly, 2000 cells per well were seeded into 96-well plates. After 48 h, the treatment was terminated. The MTS reagent (Applied Biosystems, Foster City, CA, USA) was diluted to a final concentration of 10% in phosphate-buffered saline (PBS), and 100 µL were added per well. After 1 h, the optical density (OD) was determined at 490 nm.

### Lactate dehydrogenase (LDH) release

5000 cells were inoculated into a 96 well cell culture plate and treated with drugs for 48 h. The LDH release reagent provided by the LDH cytotoxicity test kit (#C0016, Beyotime, Shanghai, China) was added in an amount of 10% of the original volume of the culture medium. The cells were further cultured for 1 h. After centrifugation at 400*g* for 3 min, 125 μL of supernatant was measured at 490 nm for each group.

### Clone formation assay

Cells were seeded into a 6-well plate at a density of 2000 cells per group. Routine cultivation lasted for approximately 10 day and was then terminated. The culture medium was discarded, 1 mL of methanol was added, and the cells were fixed for 9 min. Subsequently, 1 mL of crystal violet solution was added. After 40 min of staining, the orifice plate was washed with water and the colonies were counted.

### Scratch healing experiment

When the cells in the culture dish were full, a straight line was drawn between the dense cells with a 200 µL pipette tip. Floating cells were washed away with PBS. The specified concentration of drug were added, and the scratch width was photographed and measured at 24, 48, and 72 h.

### Transwell cell migration and invasion experiment

First, 1 × 10^5^ cells were grown in serum-free DMEM. For invasion experiment, 100 µL matrix gel was added to the upper layer of the chamber, and the well plate was placed in the cell culture incubator for 30 min. Subsequently, the cells were seeded into the upper layer of the chamber. Medium containing 20% fetal bovine serum was prepared and 650 μL of the medium were added to the bottom layer of the chamber. The cells were exposed to the drugs for 12 h and the supernatant was then slowly aspirated. Cells were fixed in methanol for 8 min and stained with crystal violet for 40 min. The excess staining solution in the mesh was gently wiped off while avoiding removing the cells. The chambers were placed under a microscope, and the cells were photographed and counted.

### Real-time quantity-PCR (qRT-PCR)

Firstly, RNAzol (MRC, Cincinnati, OH, USA) was used to extract total RNA from each group of cells. According to the instructions of the M-MLV Reverse Transcriptase kit (Sigma Aldrich), RNA was transcribed into cDNA. To remove genomic DNA, the reverse transcription reaction needed to run at 42 ℃ for 2 min. Next, reverse transcription was performed at 25 ℃, 55 ℃, and 85 ℃, respectively. According to SYBR^®^ Green PCR Kit (Sigma Aldrich) operation instructions, PCR reaction was carried out step by step. Finally, the samples were tested on the Mastercycle ep realplex detection system (Eppendorf, Hamburg, Germany). The primer sequences are shown in Table 1.

**Table 1:** Primers for qRT-PCR in this study

Gene	Primer sequence
SLC7A11	Forward: 5'-TGGTTGCCCTTTCCCTCTATTC-3' Reverse: 5'-ATGGCTGGACCTCCTAGAGTG-3'
GPX4	Forward: 5'-GAGGCAAGACCGAAGTAAACTAC-3' Reverse: 5'-CCGAACTGGTTACACGGGAA-3'
ACSL4	Forward: 5'-CATCCCTGGAGCAGATACTCT-3' Reverse: 5'-TCACTTAGGATTTCCCTGGTCC-3'
GAPDH	Forward: 5'-GGACCTGACCTGCCGTCTAG-3' Reverse: 5'-TAGCCCAGGATGCCCTTGAG-3'

### Western blotting

SLC7A11 (#26864-1-AP), GPX4 (#67763-1-Ig), and ACSL4 (#22401-1-AP) antibodies were purchased from Proteintech (Rosemont, IL, USA). Using the Micro BCA Protein Assay Kit (#23235, Thermo Fisher Scientific, Waltham, MA, USA), the protein concentration of each group was adjusted as indicated. Polyacrylamide gel electrophoresis was performed at 120 V for 90 min and 300 mA for 1 h. After transferring to a polyvinylidene difluoride membrane, it was blocked with 5% skim milk for 2 h. Subsequently, the membrane was incubated with the primary antibody (1:2000) for 9 h. Next, the secondary antibody dilution was prepared and the membrane was incubated at 23 ℃ and slowly shaken with the solution for 40 min. After washing the membrane with PBS/0.1% Tween, the chemiluminescence signals were detected.

### Detection of the Fe^2+^ content

Briefly, 3 × 10^7^ cells were resuspended in 100 µL of the iron assay buffer, followed by ultrasonic disruption for 1 h and immediate centrifugation (10,000×*g*). The supernatant was added to a 96-well plate, and 100 µL of the iron probe (#ab83366, Abcam, Cambridge, UK) were added. After incubation at 37 ℃ for 1 h, the OD value was determined at 593 nm.

### Detection of the malondialdehyde (MDA) content

The MDA reagent kit (#S0131S, Beyotime, Shanghai, China) was used to detect the malondialdehyde content. Briefly, 2 × 10^7^ cells were seeded per group, and the protein concentration was determined for each group using the Micro BCA Protein Assay Kit. Proteins from each group of cells were collected. 200 µL of the working solution from the MDA reagent kit were added to the collected protein, the sample was heated at 100 ℃ for 15 min, and then cooled in a water bath to 37 ℃. After centrifugation (10,000×g), the OD value of 200 µL of the supernatant was determined at 532 nm.

### Detection of the glutathione (GSH) content

Briefly, 2 × 10^7^ cells were seeded per group, and the protein removal solution was added to the cells at a three fold volume. The cells were alternately placed in liquid nitrogen and warm water rapidly, 3–5 times, before centrifugation (10,000×g). The reagent kit (# S0053, Beyotime, Shanghai, China) was used to detect the content of reduced glutathione. After following the manufacturer’s instructions, the total content of glutathione and oxidized glutathione disulfide were measured separately, and the reduced glutathione content was obtained by subtracting the two. The OD value was determined at 405 nm.

### si RNA transfection

genOFF st-h-PLK1_001 (#stB0005005A-1-5) was purchased from RIBOBIO (China). Opti-MEM^™^ (50 µL) (#11058021, Gibco) was mixed with 4 µL of Lipofectamine^™^ 2000 (#11668019, Gibco) and left for 5 min. Next, 50 µL of Opti-MEM^™^ was mixed with 5 µL of siRNA. The two tubes were combined and left standing for 25 min before the solution was transferred to the cell culture dish.

### Statistical analyses

Experimental data were analyzed using Prism software v. 8.0.2 (GraphPad Software, San Diego, CA, USA). Comparisons between the means of any two groups were performed using an independent-sample two-tail unpaired Student’s *t*-test. Statistical significance was set at P < 0.05.

## Results

### The combination of BI 2536 and erastin inhibits the proliferation, invasion, and migration of HNSCC cells

**Fig. 1:**
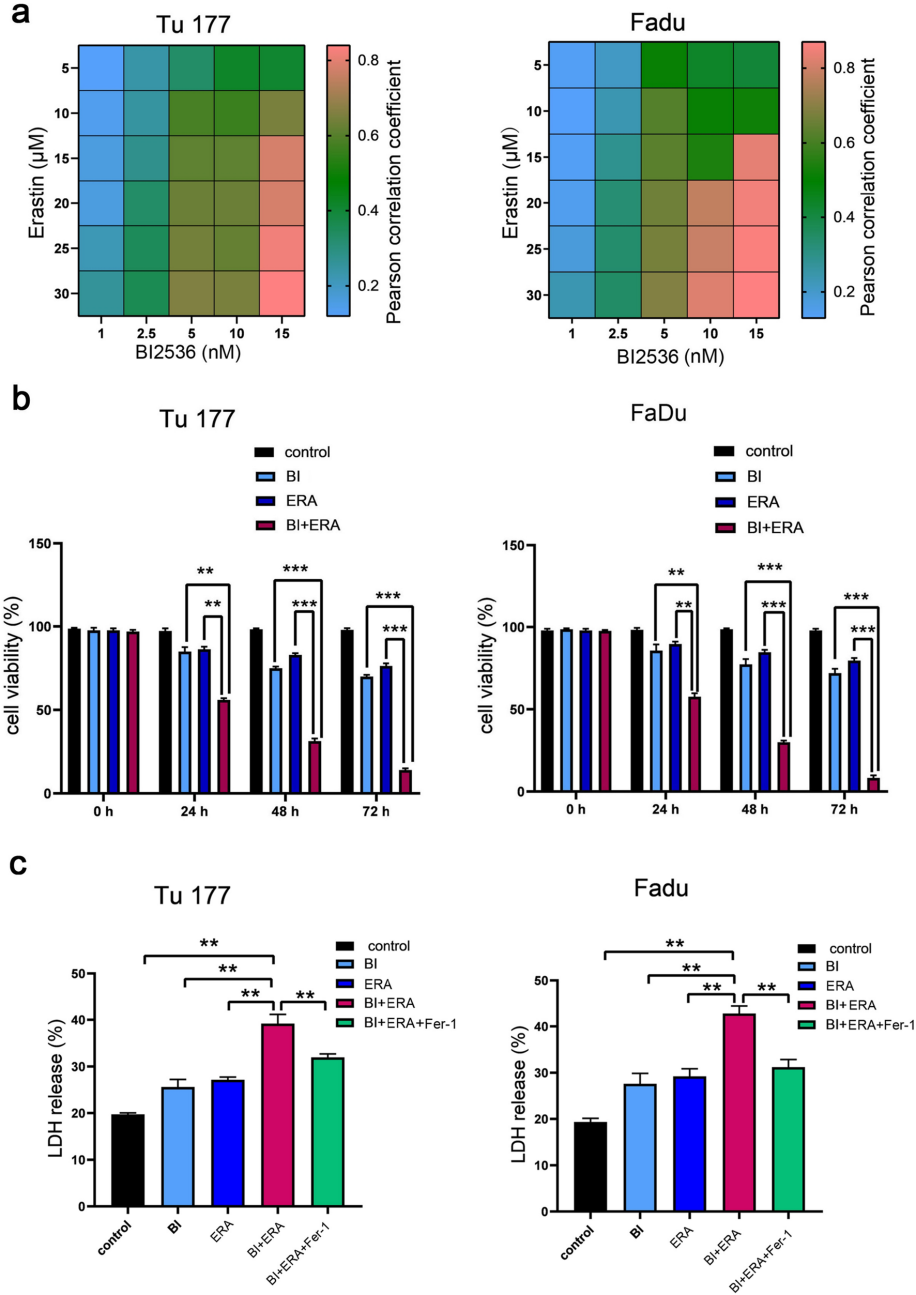
BI 2536 in combination with erastin inhibits Tu177 and FaDu cell viability. **a** The heatmaps of the changes in cell viability after co-treatment with BI2536 and erastin at different concentrations. **b** Cell viability at different time points after co-treatment with BI 2536 (5 nM) and erastin (10 μM) at the indicated concentrations. **c** Detection of cytotoxicity after the BI2536 (5 nM) and erastin (10 μM) combination for 48 h. **P < 0.01, ***P < 0.001

**Fig. 2:**
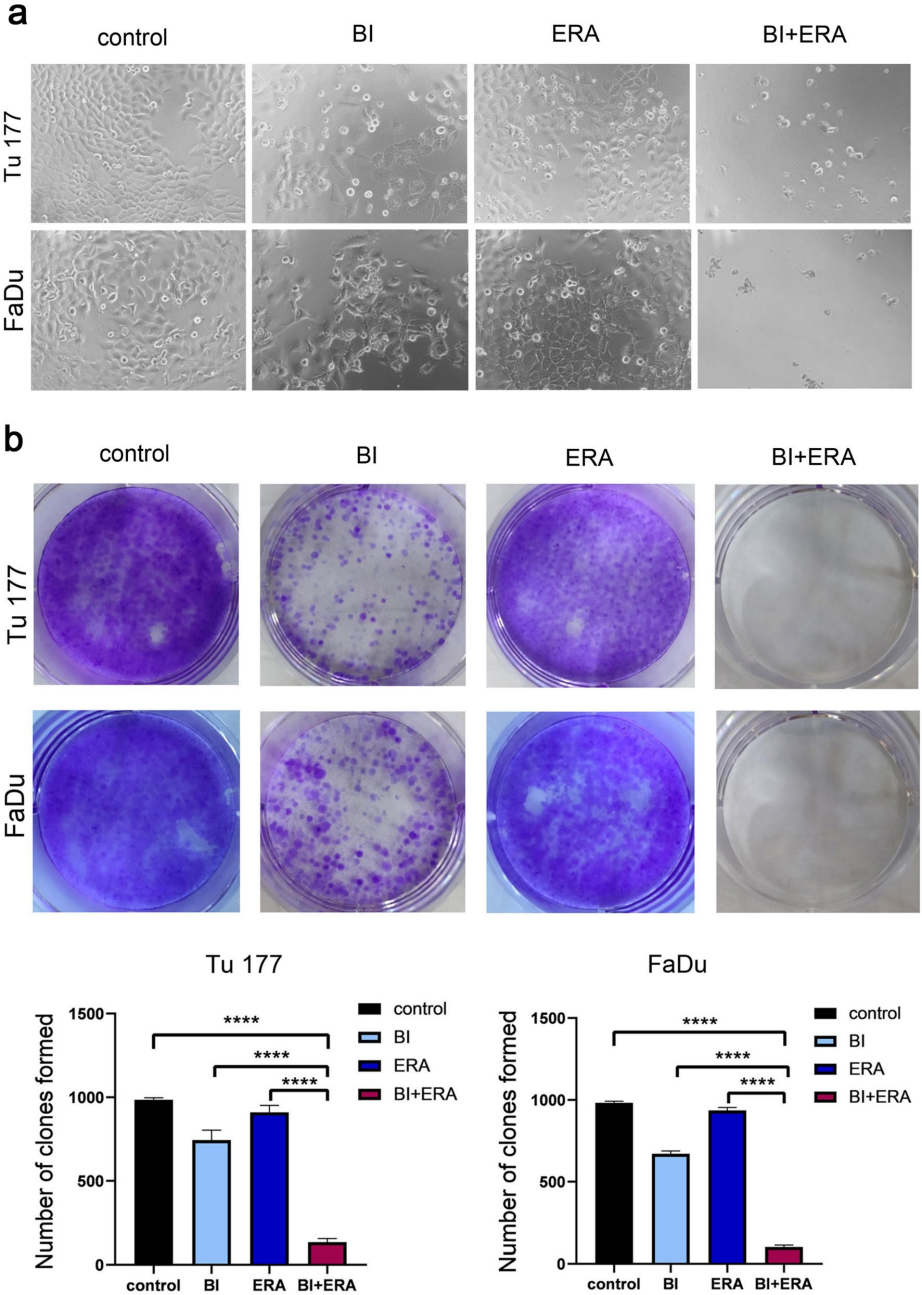
BI 2536 combined with erastin inhibits Tu177 and FaDu cell clone formation. Cells were treated with BI 2536 (5 nM) and erastin (10 μM) for 48 h. The control group was treated with DMSO. **a** Cell growth status under different conditions. **b** The state of cell clone formation. ****P < 0.0001

**Fig. 3:**
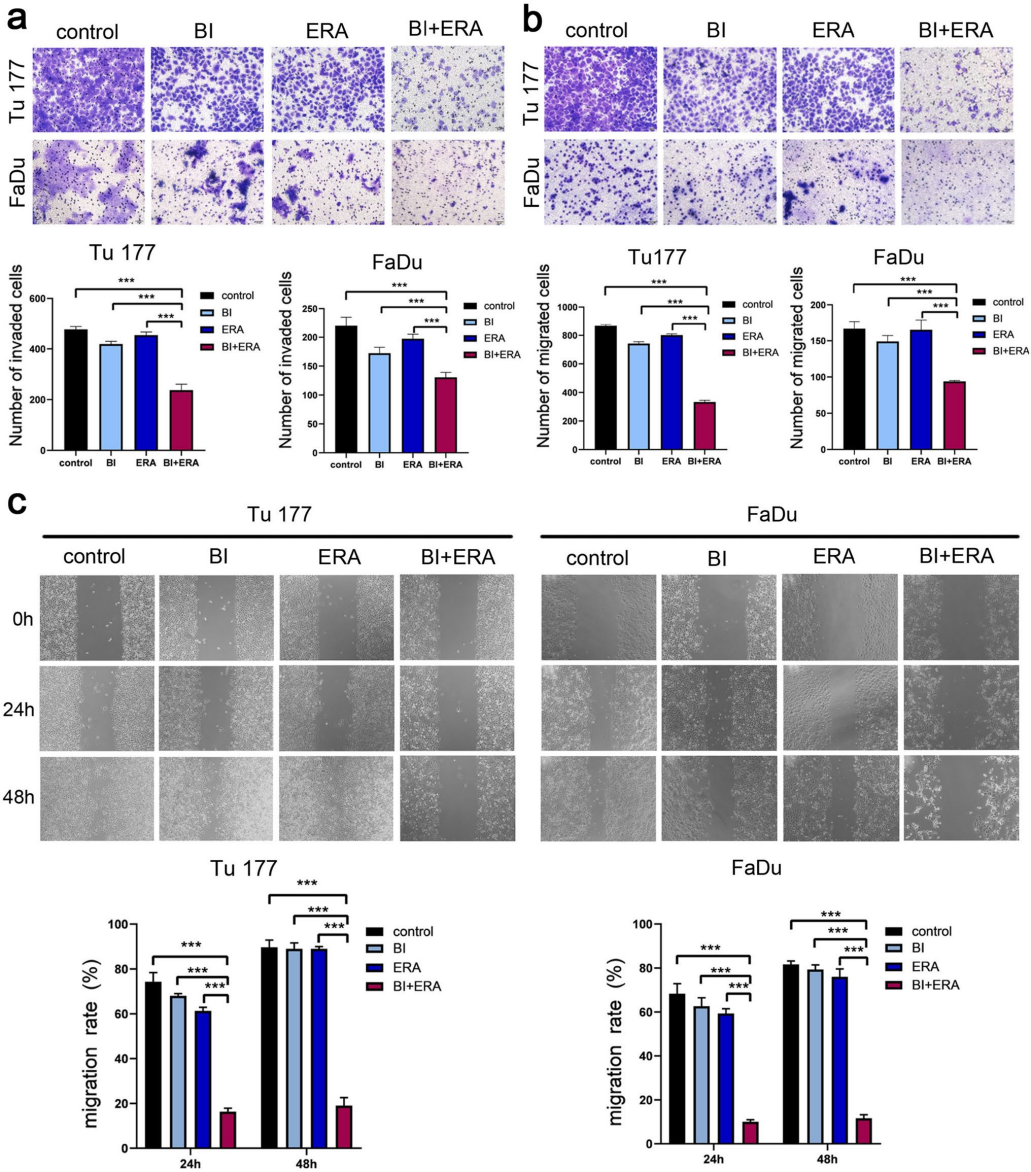
BI 2536 combined with erastin inhibits Tu177 and FaDu cell invasion and migration. Cells were treated with BI 2536 (5 nM) and erastin (10 μM) for 48 h. The control group was treated with DMSO. **a** Change in cell invasiveness (scale bar = 50 μm). **b** Change in cell migration ability (scale bar = 50 μm). **c** Comparison of cell migration rates (scale bar = 50 μm). ***P < 0.001

To evaluate the cytotoxic effects of BI 2536 (BI) and erastin (ERA), Tu177 and FaDu cells were treated in combination with BI 2536 and erastin at different concentrations for 48 h. Based on these results, BI 2536 (5 nM) and/or erastin (10 μM) were then used to treat cells for 24, 48, and 72 h. Combining the two drugs exerted a stronger inhibitory effect on cell viability than treatment with a single agent (Fig. 1a–c). Moreover, cytotoxicity could be reversed by the ferroptosis inhibitor, ferrostatin-1 (Fer-1) (15 μM) (Fig. 1c). Subsequent experiments demonstrated that the combination of BI 2435 and erastin significantly inhibited the self-renewal ability of tumor cells (Fig. 2a–b). Correspondingly, the invasion and migration abilities of cells were significantly weakened (Fig. 3a–c).

### Combined treatment with BI 2536 and erastin enhances Fe^2+^ and lipid peroxide accumulation, and GSH consumption

**Fig. 4:**
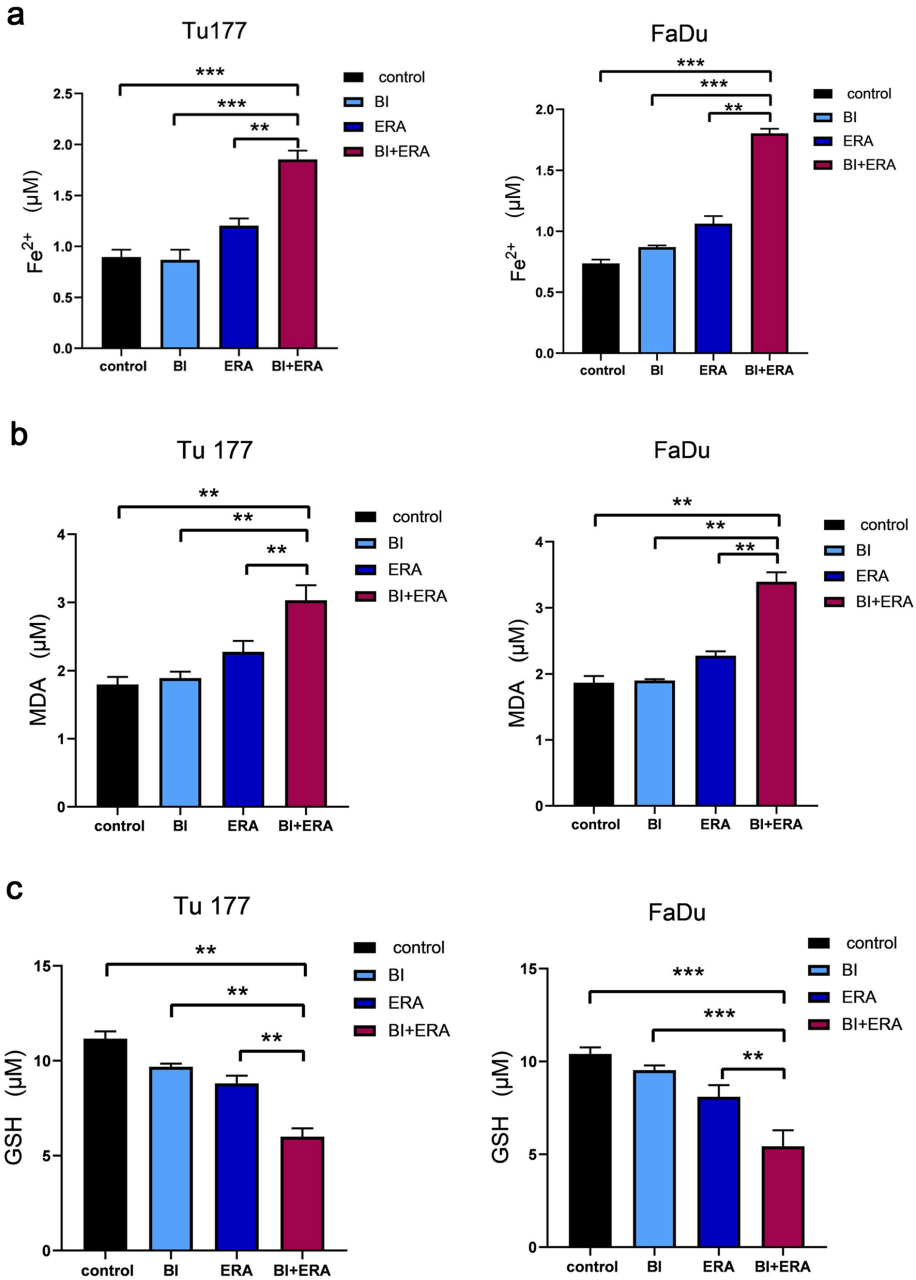
Combining BI 2536 and erastin enhances the degree of ferroptosis. Tu177 and FaDu cells were treated with BI 2536 (5 nM) and erastin (10 μM) for 48 h. The control group was treated with DMSO. **a** Change in Fe^2+^ content. **b** Change in MDA content. **c** Change in GSH content. **P < 0.01,***P < 0.001

Because erastin is a classic inducer of ferroptosis [24], we evaluated whether BI 2536 enhanced its ferroptosis-inducing effect. The results showed that, compared to a single treatment, combining the two agents resulted in a greater accumulation of Fe^2+^ and MDA, along with severe depletion of GSH (Fig. 4a–c).

### Combined treatment with BI 2536 and erastin enhances alterations in ferroptosis-related gene expression

**Fig. 5:**
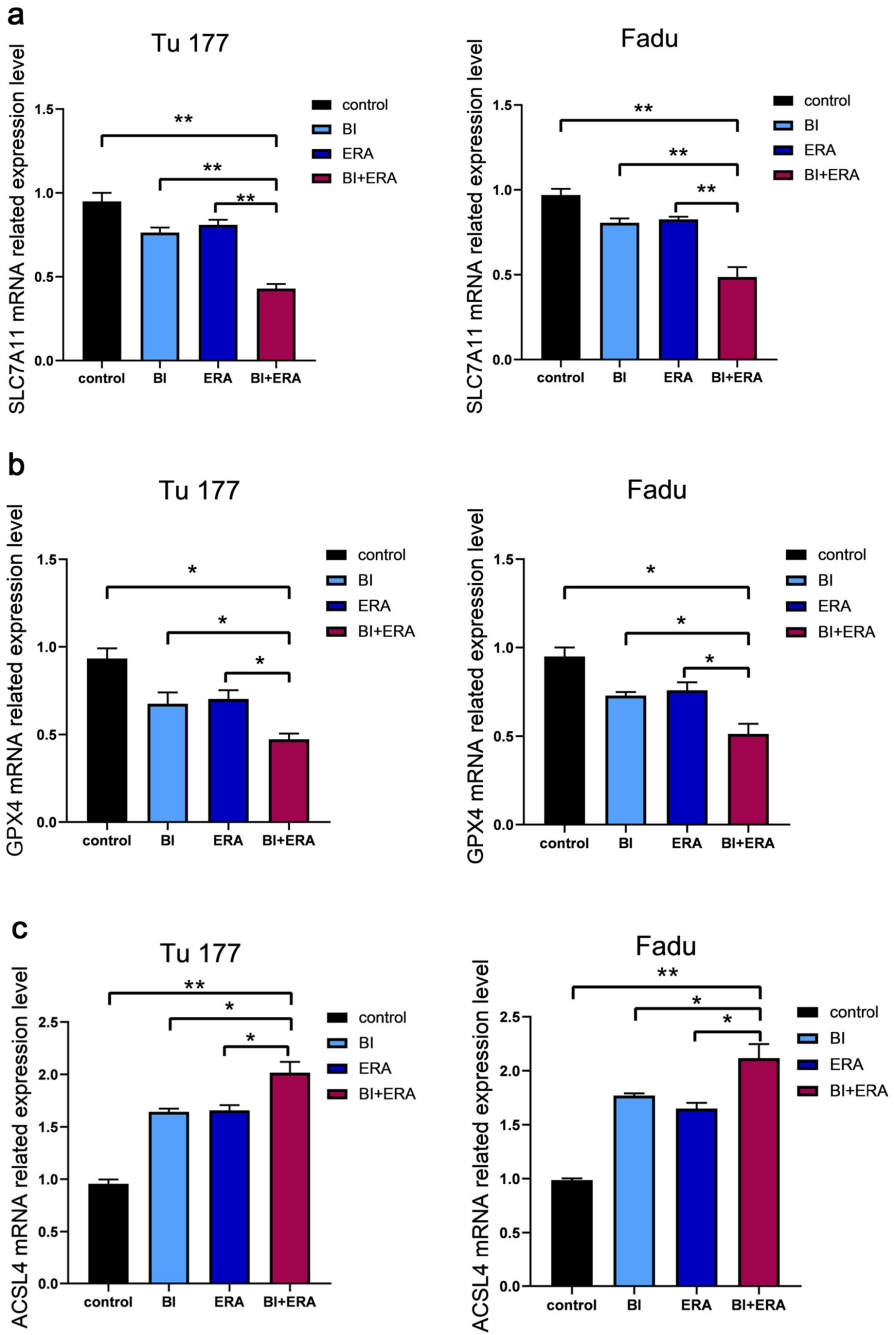
Combining BI 2536 and erastin enhances changes in the mRNA level of ferroptosis genes. Detection of SLC7A11 (**a**), GPX4 (**b**), ACSL4 (**c**) mRNA with BI 2536 (5 nM) and erastin (10 μM) for 48 h. The control group was treated with DMSO. *P < 0.05, **P < 0.01

**Fig. 6:**
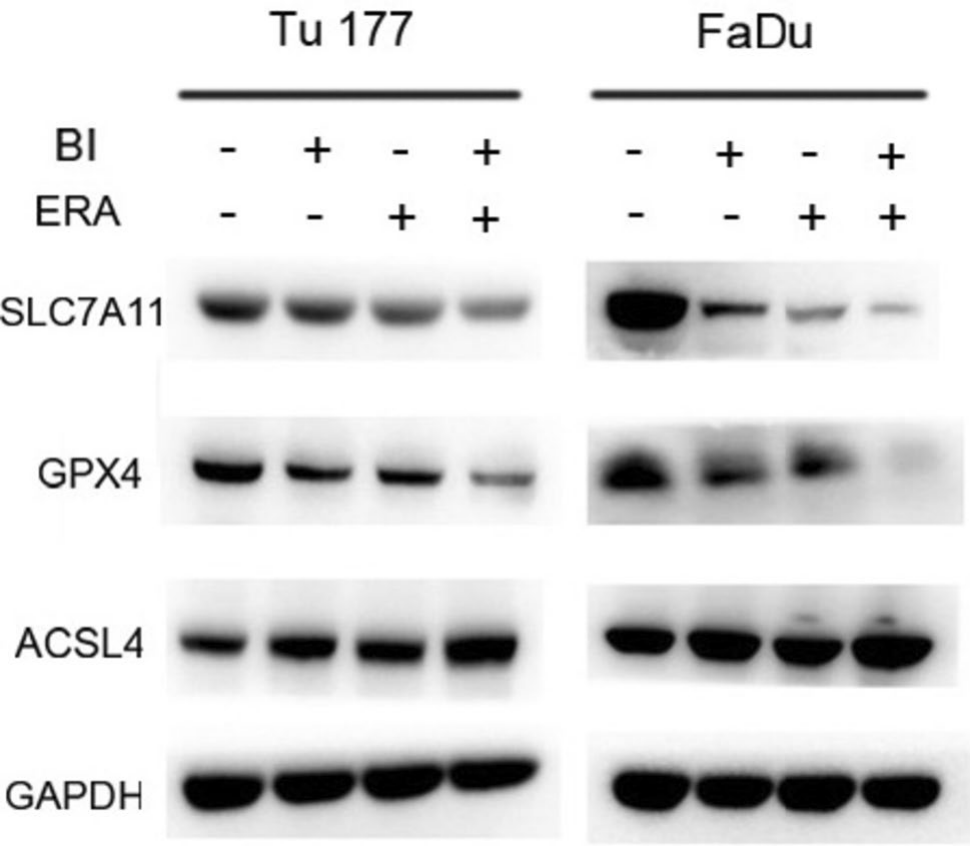
Combining BI 2536 and erastin enhances changes in the expression of ferroptosis genes. Expression of ferroptosis genes after treatment of Tu177 and FaDu cells with BI 2536 (5 nM) and erastin (10 μM) for 48 h

After observing the enhanced ferroptosis by the combined BI 2536 and erastin treatment, the mRNA and expression changes of ferroptosis-related genes were further determined. This combination resulted in lowered levels of SLC7A11 and GPX4 mRNA and proteins, and accumulation of the ACSL4 mRNA and protein (Figs. 5a–c and 6).

### Targeting PLK1 has a synergistic effect with erastin

**Fig. 7:**
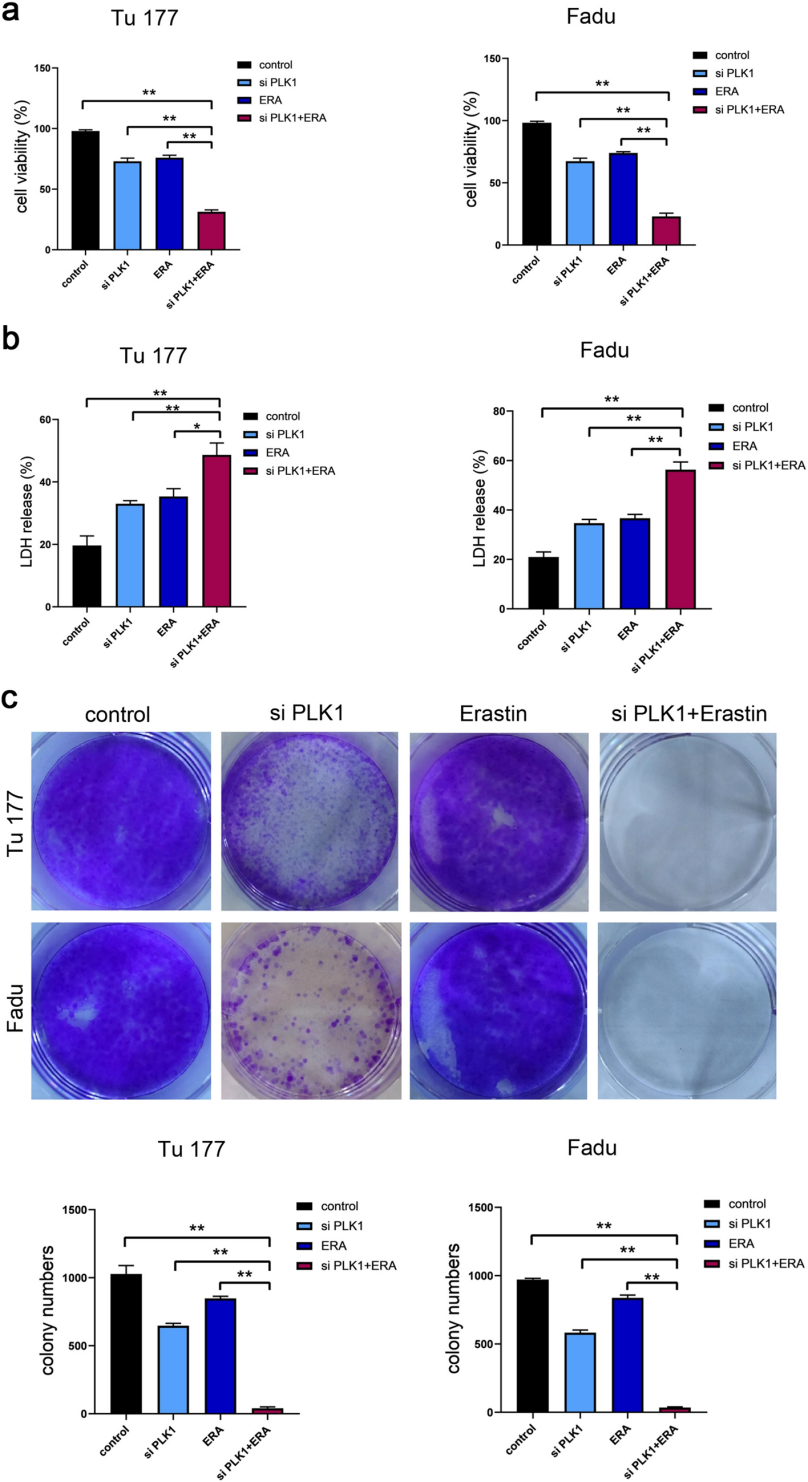
Targeting PLK1 in combination with erastin inhibits cell proliferation. Tu177 and FaDu cells were treated with si PLK1 (5 nM) and erastin (10 μM) for 48 h. The control group was treated with DMSO. **a** Changes in cell viability. **b** Detection of cytotoxicity. **c** Changes in the ability of cells to form clones. *P < 0.05, **P < 0.01

**Fig. 8:**
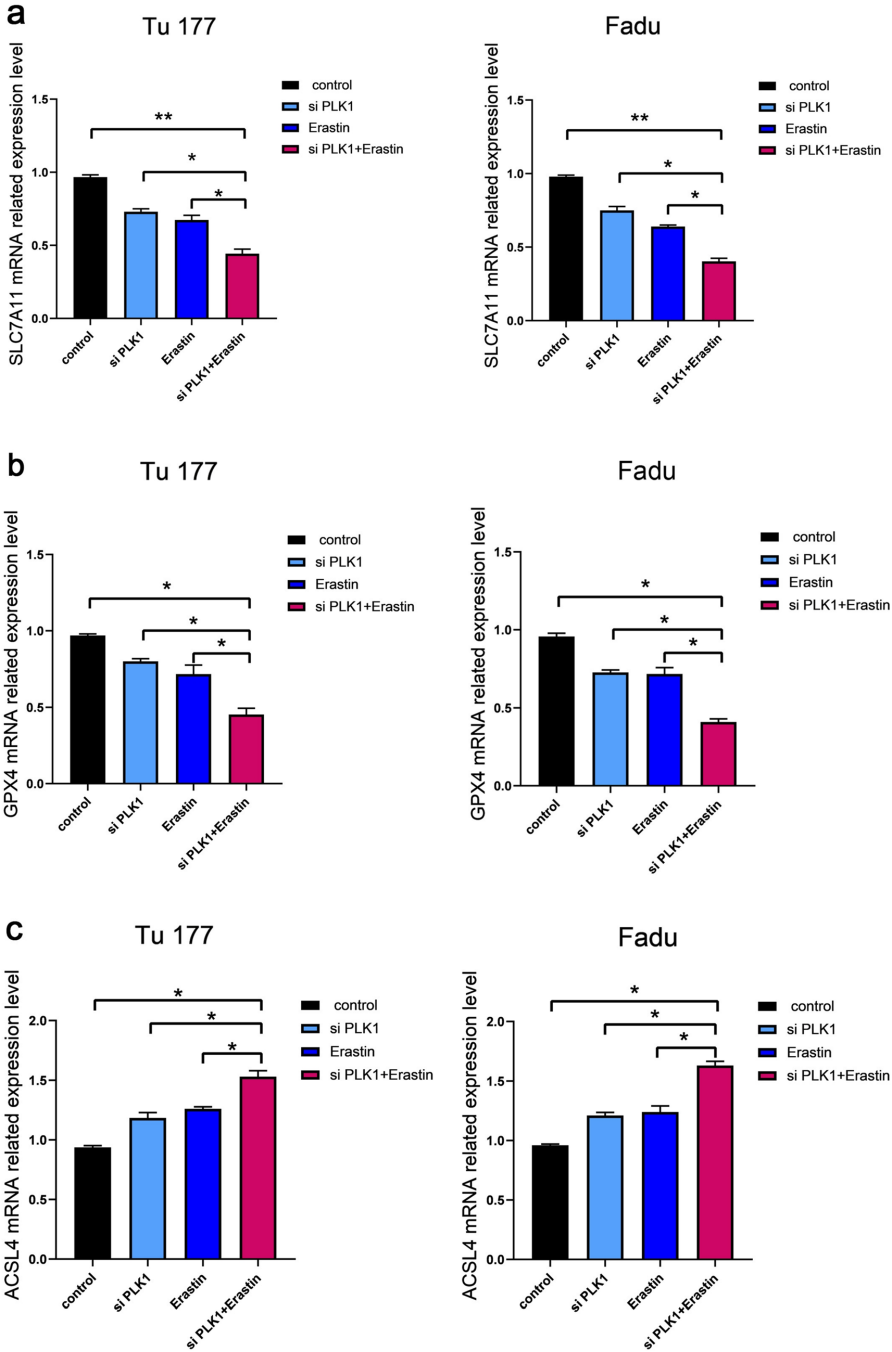
Targeting PLK1 promotes erastin induced ferroptosis. Detection of SLC7A11 (**a**), GPX4 (**b**), ACSL4 (**c**) mRNA with si PLK1 (5 nM) and erastin (10 μM) for 48 h. The control group was treated with DMSO. *P < 0.05, **P < 0.01

To further verify that targeting PLK1 can promote erastin-induced ferroptosis, the interfering RNA targeting PLK1 was used in subsequent experiments. The consistent results showed that under the dual effects of the both, the cell proliferation ability was significantly weakened (Fig. 7a), the cytotoxicity was enhanced (Fig. 7b) and the self-renewal ability was enhanced (Fig. 7c). Moreover, the mRNA content of SLC7A11 and GPX4 decreased, while the mRNA content of ACSL4 increased (Fig. 8a–c).

## Discussion

Ferroptosis, a regulated form of cell death, is completely different from other forms of cell death. Specifically, it is accompanied by a large accumulation of iron and lipid peroxides [25]. Accumulating evidence suggests that inducing ferroptosis may be a feasible strategy for treating cancer, as triggering this form of cell death can effectively kill tumor cells resistant to pro-apoptotic and other conventional anti-tumor therapies [26, 27]. However, there are differences in sensitivity to ferroptosis among different types of tumors and patients [13]. Currently, the signaling pathways and influencing factors that make tumor cells sensitive to ferroptosis are not fully explored, and the issue of insufficient sensitivity of some tumors to ferroptosis inducers needs to be addressed.

Ferroptosis inducers can be divided into two types. The first type, including erastin and sulfasalazine, works by acting on cystine glutamate reverse transporters (system Xc^−^) [27]. The second type, including RSL3, acts directly on GPX4 to inhibit its activity [27, 28]. The difference between erastin and other inducers is that the latter usually only activate a single signaling pathway, while erastin acts on multiple molecular structures with good, rapid, and long-lasting effects, and its preclinical efficacy has been demonstrated preliminarily [29]. From the perspective of developing the therapeutic effect of ferroptosis inducers, we combined the inhibition of PLK1 with ferroptosis. According to literature search, there is currently no relevant research on BI2536 in the field of ferroptosis. Here, we are the first to confirm the synergistic effect between BI2536 and erastin.

In this study, compared to using erastin alone, BI 2536 further increased the degree of intracellular Fe^2+^ accumulation. ACSL4 is a key molecule that regulates lipid composition, mainly by catalyzing membrane phospholipid oxidation to regulate fatty acid metabolism [6]. In this study, under the simultaneous action of BI 2536 and erastin, ACSL4 expression increased, and MDA production increased, indicating that BI 2536 may increase erastin-induced lipid peroxidation by upregulating ACSL4. A stable antioxidant system can prevent cell ferroptosis, and the system Xc^−^/GSH/GPX4 axis is the core link in regulating redox homeostasis [9]. In the present study, BI 2536 may exacerbate erastin-induced GSH depletion by inhibiting the expression of SLC7A11 and GPX4. Therefore, ACSL4, SLC7A11, and GPX4 are key links necessary links in the synergistic effect between these two, providing an experimental basis for HNSCC treatment with ferroptosis inducers and PLK1 inhibitors. Although these pathways are generally important for maintaining survival in non-cancer cells and tissues, regulating key ferroptosis checkpoints may be sufficient to make cancer cells more highly susceptible to this form of cell death [13].

PLK1 is the earliest discovered and most characteristic member of the Ser/Thr kinase family [30]. Recently, in addition to PLK1 being a promising drug development target, many other potent selective RNA polymerase II transcriptional related kinase inhibitors have also been developed, such as cyclin dependent kinases (CDKs) inhibitors [31]. Given that both the CDKs family and PLK1 control tumor cell survival and progression by regulating cell cycle, a reasonable speculation is that CDKs inhibitors may also have an enhanced ferroptosis inducers sensitivity effect similar to BI2536. Thus, the combination of kinase inhibitors and ferroptosis inducers may provide a new direction for tackling tumors in the future.

The study of cell death pathways has opened up new avenues for cancer treatment; however, multiple mechanisms of ferroptosis, and the sensitivity of tumor cells to ferroptosis, are still being studied and updated, consequently raising more and new questions. In summary, the results of this study reveal that the PLK1 inhibitor, BI 2536, has broad therapeutic prospects in HNSCC, especially when combined with a ferroptosis inducer such as erastin. Nevertheless, studies in vivo are needed in the future to validate the sensitization effect. The current evidencein vitro provides new insights for combination therapy and may help improve tumor treatment strategies by inducing ferroptosis.

## Data Availability

All data are available in the main text or available upon request to the corresponding author.
